# Interferon-Associated Inflammatory Remodeling and Oxidative Metabolic Repression Define the Skeletal Muscle Transcriptomic Landscape of δ-Sarcoglycan Deficiency

**DOI:** 10.3390/biom16101511

**Published:** 2026-10-17

**Authors:** Amairani Cancino-Bello, Mauricio Hernández-Somilleda, Erick Bahena-Culhuac, Estela G. García-González, Oscar Hernández-Hernández, Ramón M. Coral-Vázquez, J. Manuel Hernández-Hernández

**Affiliations:** 1 Laboratorio de Epigenética de la Regeneración Muscular, Departamento de Genética y Biología Molecular, Centro de Investigación y de Estudios Avanzados (CINVESTAV-IPN), Mexico City 07360, Mexico; amairani.bello@cinvestav.mx (A.C.-B.); mauricio.hernandezs@cinvestav.mx (M.H.-S.); erickb_2000@hotmail.com (E.B.-C.); 2 Laboratorio de Epigenética de la Regeneración Muscular, Centro de Investigación sobre el Envejecimiento, Centro de Investigación y de Estudios Avanzados (CINVESTAV-IPN), Mexico City 14330, Mexico; estela.garcia@cinvestav.mx; 3 Laboratorio de Medicina Genómica, Departamento de Genética, Instituto Nacional de Rehabilitación–Luis Guillermo Ibarra Ibarra, Mexico City 14389, Mexico; heroscar@gmail.com; 4 Subdirección de Enseñanza e Investigación, Centro Médico Nacional 20 de Noviembre, Instituto de Seguridad y Servicios Sociales de los Trabajadores del Estado, Mexico City 03100, Mexico; rmcoralv@gmail.com; 5 Sección de Estudios de Posgrado e Investigación, Escuela Superior de Medicina, Instituto Politécnico Nacional, Plan de San Luis y Díaz Mirón s/n, Col. Casco de Santo Tomas, Mexico City 07700, Mexico

**Keywords:** RNA-seq, limb-girdle muscular dystrophy, LGMD2F, δ-sarcoglycan, Sgcd, skeletal muscle, interferon response, innate sensing, macrophages, oxidative metabolism, (-)-epicatechin

## Abstract

Limb-girdle muscular dystrophy R6, formerly LGMD2F, is caused by mutations in the δ-sarcoglycan (*Sgcd*) gene and is characterized by progressive skeletal muscle degeneration. Although loss of δ-SG protein compromises sarcolemma stability, how δ-sarcoglycan deficiency reshapes inflammatory and metabolic gene programs across distinct skeletal muscles remains incompletely defined. Here, we profiled soleus and extensor digitorum longus (EDL) muscles from wild-type and *Sgcd*^−/−^ mice treated with vehicle or (-)-epicatechin, a dietary flavanol with anti-inflammatory and antioxidant properties, using bulk RNA sequencing, pathway analysis, immune-cell deconvolution, and RT-qPCR validation. δ-sarcoglycan deficiency produced broad transcriptional remodeling while preserving baseline muscle-type identity. Across soleus and EDL, the dystrophic transcriptome was marked by interferon-associated inflammatory remodeling together with repression of oxidative and mitochondrial metabolic programs. Within this shared disease state, soleus displayed a stronger induced inflammatory component, including innate-sensing and macrophage-linked features. Computational deconvolution, which estimates immune-cell proportions from bulk transcriptomes (quanTIseq and mMCP-counter), supported genotype-dependent shifts in immune-cell signatures, with concordant monocyte/macrophage signals. Finally, (-)-epicatechin produced context-dependent transcriptional effects and attenuated a defined interferon/innate-sensing module in dystrophic soleus, as supported by RNA-seq and RT-qPCR. Together, these findings establish inflammatory remodeling with a prominent interferon component and oxidative metabolic repression as central transcriptional features of δ-sarcoglycan-deficient skeletal muscle, while revealing an amplified innate-sensing and immune-enriched module in dystrophic soleus that remains transcriptionally responsive to (-)-epicatechin treatment.

## 1. Introduction

Limb-girdle muscular dystrophy type 2F, now classified as limb-girdle muscular dystrophy R6 (LGMD-R6), is a recessive muscular dystrophy caused by mutations in the delta-sarcoglycan gene (SGCD) [1]. δ-sarcoglycan is an essential component of the dystrophin-associated protein complex, and its deficiency compromises sarcolemma stability, contributing to cycles of myofiber injury, necrosis, inflammation, fibrosis, mitochondrial stress, and progressive weakness [2]. Although the genetic basis of LGMD2F is established, the transcriptional programs that accompany disease progression in distinct skeletal muscles remain less well characterized.

Skeletal muscles differ in fiber-type composition, metabolic profile, contractile demand, and susceptibility to injury, and these intrinsic differences can shape how dystrophic pathology develops across muscle groups. In Duchenne muscular dystrophy, fast fibers and specific muscle groups are preferentially vulnerable [3]. This fiber-type-dependent susceptibility is particularly evident in the myotonic dystrophies. Type 1 myofibers are preferentially affected in myotonic dystrophy type 1 (DM1), while type 2 myofibers are affected in DM2 [4]. These observations indicate that fiber-type identity can determine which muscles are most affected by dystrophic damage, providing a framework for interpreting muscle-specific vulnerability in other dystrophies.

The *Sgcd*^−/−^ mouse is a well-established model of LGMD2F that reproduces key features of the human disease, including progressive muscle degeneration and cardiomyopathy [5]. In this model, pathological remodeling varies across skeletal muscles: increased collagen deposition has been reported in the slow-twitch soleus compared with other skeletal muscles [6], and dysregulated ATX-LPA/YAP-TAZ signaling has been described in triceps, gastrocnemius, and diaphragm, linking lipid-mediated signaling with early inflammation and fibrosis [7]. Together, these observations suggest that the transcriptional response to dystrophic injury differs between muscles of distinct fiber-type composition, but the pathways underlying these fiber-type-biased responses remain incompletely understood.

Chronic inflammation is a prominent feature of muscular dystrophies and contributes to disease progression through persistent cytokine signaling, immune-cell recruitment, myofiber damage, and tissue remodeling. In Duchenne muscular dystrophy, persistent activation of IKK/NF-κB signaling in both immune cells and muscle fibers drives chronic inflammation, fibrosis, and muscle degeneration, and its inhibition improves dystrophic pathology [8]. In sarcoglycanopathies, transcriptomic and immune-cell-associated signatures have been linked to disease severity and inflammatory heterogeneity [9]. Beyond these cytokine-driven programs, a distinct arm of innate immunity involves the sensing of endogenous nucleic acids: damaged myofibers can release damage-associated molecular patterns, including self-derived RNA, that are detected by pattern-recognition receptors such as Toll-like receptors and cytosolic RIG-I-like receptors (RLRs; RIG-I, MDA5, and LGP2). Activation of these sensors engages interferon regulatory factors alongside NF-κB, driving type-I interferon production and the induction of interferon-stimulated genes [10,11]. This nucleic-acid sensing/interferon axis has been directly implicated in muscle disease: type-I interferon and Toll-like receptor expression characterize inflammatory myopathies [12], and in myotonic dystrophy, the accumulation of double-stranded RNA activates the innate immune response and interferon signaling [13]. Whether a comparable axis operates in sarcoglycanopathies, and how it is distributed across metabolically distinct muscles, remains unknown. In addition, whether distinct muscles in LGMD2F differ primarily in the extent, composition, and immune-metabolic context of inflammatory remodeling, and whether the nucleic-acid sensing/interferon axis contributes to this heterogeneity, has not been systematically examined.

In addition to defining disease-associated transcriptional remodeling, there is interest in identifying interventions that modulate inflammatory and stress-associated pathways in dystrophic muscle. (-)-Epicatechin, a naturally occurring flavanol abundant in cocoa-derived products and detected in other plant-derived sources, including apples and tea [14,15,16], has anti-inflammatory and antioxidant properties and has been reported to influence vascular and metabolic function, myogenic differentiation, and skeletal muscle-related outcomes in several experimental settings [17,18,19,20,21]. In dystrophic muscle, it has been shown to promote mitochondrial biogenesis and improve muscle-related outcomes [22]. However, its transcriptional effects in δ-sarcoglycan-deficient skeletal muscle have not been evaluated.

Here, we analyzed bulk RNA-seq profiles from soleus and EDL muscles of wild-type and *Sgcd*^−/−^ mice treated with vehicle or (-)-epicatechin. By combining differential expression, GO and KEGG pathway analysis, immune-cell deconvolution, and RT-qPCR validation, we sought to define how δ-sarcoglycan deficiency reshapes inflammatory, innate-sensing, and metabolic transcriptional programs across distinct skeletal muscles. We further evaluated whether (-)-epicatechin treatment can reshape the disease-associated transcriptional state of dystrophic muscles.

## 2. Materials and Methods

### 2.1. Chemicals

(-)-Epicatechin was obtained from Sigma-Aldrich (St. Louis, MO, USA) and prepared for oral administration, as previously described [19], using 1% DMSO Sigma-Aldrich (St. Louis, MO, USA) diluted in water as vehicle.

### 2.2. Animals

Wild-type (WT; B6.129-*Sgcd*) and δ-sarcoglycan knockout (*Sgcd*^−/−^; B6.129-*Sgcd*tm1Mcn/J) male mice, 8–12 weeks old at the start of treatment, were housed in groups under controlled environmental conditions (12 h light/dark cycle, 22 ± 2 °C, 50–60% relative humidity). No specific acclimatization period was applied prior to the start of the procedures. The *Sgcd*^−/−^ mouse model has been previously described [23], and the original strain was obtained from the Jackson Laboratory (Bar Harbor, ME, USA; strain #004582). Genotypes were confirmed according to the Jackson Laboratory protocol. All experimental procedures were conducted in accordance with institutional guidelines (CICUAL, UPEAL 0400-24) and the Mexican Official Norm (NOM-062-ZOO-1999).

### 2.3. Experimental Design and Treatment

The transcriptional effects of δ-sarcoglycan deficiency and (-)-epicatechin treatment were evaluated in soleus and EDL muscles from WT and *Sgcd*^−/−^ mice. Four experimental groups were analyzed: vehicle-treated WT, (-)-epicatechin-treated WT, vehicle-treated *Sgcd*^−/−^, and (-)-epicatechin-treated *Sgcd*^−/−^. Within each genotype, mice were randomly assigned to the vehicle or (-)-epicatechin treatment group. Animals showing signs of fighting-related injury were excluded from the study. No blinding was performed, as group identity was required for the subsequent transcriptomic comparisons. Mice received vehicle (1% DMSO diluted in water) or (-)-epicatechin (1 mg/kg body mass) by oral gavage twice daily for 15 consecutive days. On day 16, mice were euthanized by cervical dislocation, and soleus and EDL muscles were dissected and preserved in RNAlater™ (Thermo Fisher Scientific Baltics UAB, Vilniaus, Lithuania) at −80 °C until processing.

### 2.4. RNA Isolation and Bulk RNA-seq

For each experimental group, soleus or EDL muscles from two mice were pooled to constitute one biological replicate, yielding three pooled biological replicates per condition. The pooled sample (two mice) constituted the experimental unit for all analyses. The number of biological replicates (*n* = 3 per condition) was determined by the limited availability of *Sgcd*^−/−^ animals for the study. Total RNA was extracted using TRIzol^™^ (Thermo Fisher Scientific, Carlsbad, CA, USA) reagent followed by purification with the EZ-10 Spin Column Total RNA Mini-Preps kit (Bio Basic Inc., Toronto, ON, Canada). Genomic DNA was removed using DNase I (Fermentas, Stanford, CA, USA). RNA integrity was assessed using an Agilent 2100 Bioanalyzer (Santa Clara, CA, USA) with an RNA 6000 Nano Chip; all samples had RNA integrity number (RIN) values ≥ 8 and were included in library preparation. Library preparation was performed using the MGIEasy RNA Library Prep Kit (Shenzhen MGI Biological Electronic Technology Co., Ltd. Shenzhen, China), NEBNext^®^ Poly(A) mRNA Magnetic Isolation Module, and MGIEasy DNA Adapters-96 Kit (Shenzhen MGI Biological Electronic Technology Co., Ltd. Shenzhen, China). Twenty-four libraries were sequenced on the MGISeq 2000 (Shenzhen MGI Biological Electronic Technology Co., Ltd. Shenzhen, China platform using DNBSEQ^™^ technology to generate approximately 17 million single-end 100 bp reads per sample.

### 2.5. Transcriptomic Analyses

Raw sequencing quality was assessed using FastQC (v0.11.8) (https://www.bioinformatics.babraham.ac.uk/projects/fastqc/ accessed on 20 May 2025). Reads were aligned to the Mus musculus reference genome (Ensembl GRCm39) using STAR aligner (v2.7.10b) [24], and gene-level read counts were quantified using featureCounts (v2.0.3) [25]. Genes with 10 or fewer counts across all samples were filtered before analysis. All analyses were performed in R (v4.4.0). Differential expression analysis was performed using DESeq2 (v1.44.0) [26] from raw count matrices. Genes with an adjusted *p*-value ≤ 0.05 were considered differentially expressed unless otherwise specified. Jitter plots, UpSet plots, heatmaps, bar plots, dot plots, dumbbell plots, treemaps, and scatter plots were generated using ggplot2 (v4.0.0), ComplexHeatmap (v2.20.0), circlize (v0.4.16), ggpubr (v0.6.2), UpSetR (v1.4.0), and treemapify (v2.5.6).

### 2.6. Pathway Enrichment and Gene Set Analyses

Gene Ontology (GO) enrichment analysis was performed using clusterProfiler (v4.12.6) [27] with org.Mm.eg.db (v3.19.1) as the annotation database. GO Biological Process terms and KEGG pathways were analyzed separately for up- and downregulated gene sets using over-representation analysis (enrichGO and enrichKEGG). *p*-values were adjusted using Benjamini–Hochberg, with adjusted *p* < 0.05 and *q*-value < 0.2 considered significant. For gene expression heatmaps, variance-stabilized transformed expression values were row-scaled and displayed as Z-scores.

### 2.7. Immune-Cell Deconvolution

Immune-cell-associated transcriptional signatures were estimated from bulk RNA-seq data using two complementary computational deconvolution approaches applied to TPM-normalized expression matrices. mMCP-counter (mMCPcounter v1.1.0) [28] was used to estimate enrichment scores for immune and stromal cell populations in mouse tissues, using the GRCm39 genome version and gene symbol identifiers. quanTIseq (quantiseqr v1.12.0) [29] was used to estimate relative fractions of immune-cell populations, including macrophage subsets, monocytes, neutrophils, dendritic cells, NK cells, T cells, and B cells, using the TIL10 signature matrix with tumor mode disabled and mRNA scaling enabled. Concordance between the two methods was assessed by Spearman rank correlation.

### 2.8. cDNA Synthesis and Quantitative PCR

Total RNA (500 ng) from the same pooled biological replicates used for RNA-seq was reverse-transcribed using M-MLV reverse transcriptase and oligo(dT)_20_ primers (ThermoFisher Scientific, Waltham, MA, USA). Real-time PCR was carried out using SYBR Green (Applied Biosystems, Foster City, CA, USA) chemistry on the Rotor-Gene Q system (QIAGEN, Hilden, Germany), using 25 ng of cDNA per reaction. Relative expression was calculated using the comparative Ct method, with *Tbp* as the reference gene. RT-qPCR was used to validate selected inflammatory, interferon-stimulated, TLR/NF-κB pathway, and innate-sensing genes in dystrophic soleus muscle treated with vehicle or (-)-epicatechin.

### 2.9. Statistical Analysis

All statistical analyses were performed in R. Differential expression was assessed using DESeq2 with Benjamini–Hochberg-adjusted *p*-values, and genes with adjusted *p* < 0.05 were considered differentially expressed. For comparisons involving pooled biological replicates (RT-qPCR and immune-cell deconvolution scores), differences between two groups were evaluated using two-tailed Student’s *t*-tests. For comparisons of expression magnitude across gene sets between muscles, where each data point represents an individual gene measured in both tissues, paired Wilcoxon signed-rank tests were used as a conservative, non-parametric approach that is robust to potential outliers in log_2_ fold-change values. Correlations between muscles and between deconvolution methods were assessed using Spearman’s rank correlation. Bar plots show the mean with error bars representing the standard deviation (SD). Statistical significance was defined as *p* < 0.05.

## 3. Results

### 3.1. Global Transcriptomic Profiling Reveals Shared Dystrophic Remodeling and Preserved Muscle-Type Identity in δ-Sarcoglycan Deficiency

To define the transcriptional impact of δ-sarcoglycan deficiency, we analyzed bulk RNA-seq profiles from soleus and EDL muscles of WT and *Sgcd*^−/−^ mice. The *Sgcd*^−/−^ mice analyzed here were 8–12 weeks old, an age at which this model displays established muscle pathology with prominent fibrotic, inflammatory, and regenerative features [5]; the transcriptional signatures described below therefore reflect a stage of active, established disease. Principal component analysis showed that muscle identity was the major source of variance, with EDL and soleus separated along PC1, which accounted for 76% of the variance. Genotype-associated differences were captured primarily along PC2, which accounted for 20% of the variance (Figure 1A). Thus, δ-sarcoglycan deficiency substantially remodeled the transcriptome while preserving the dominant transcriptional distinction between oxidative and glycolytic muscles (Supplementary Figure S1). Consistent with this interpretation, the soleus-versus-EDL comparison performed independently within each genotype identified largely overlapping sets of muscle-enriched genes in WT and *Sgcd*^−/−^ mice, with canonical fiber-type and metabolic identity markers remaining preferentially associated with their corresponding muscle type regardless of genotype (Supplementary Figure S1B; Supplementary Table S5). This indicates that the transcriptional programs distinguishing oxidative from glycolytic muscle are largely maintained despite dystrophic remodeling. Differential expression analysis revealed broad genotype-associated transcriptional remodeling in both muscles. In soleus, *Sgcd*^−/−^ mice showed 1784 upregulated and 1186 downregulated genes relative to WT controls, whereas EDL showed 1453 upregulated and 1606 downregulated genes (Figure 1B; Supplementary Table S1). UpSet analysis demonstrated a substantial shared component of the dystrophic response, including 975 commonly upregulated genes and 712 commonly downregulated genes across soleus and EDL (Figure 1C). Consistent with this overlap, log2 fold-change values for shared differentially expressed genes were strongly correlated between muscles (Spearman rho = 0.935, *p* < 2.2 × 10^−16^; Figure 1D), indicating that much of the transcriptional response to δ-sarcoglycan deficiency is conserved in both muscles. Nevertheless, several genes showed broader effect sizes in soleus than in EDL, revealing a muscle-biased component within the otherwise shared dystrophic response.

A heatmap of representative disease-associated genes further illustrated the coordinated transcriptional separation between WT and *Sgcd^−/−^* muscles (Figure 1E). These genes included immune and inflammatory mediators, muscle regeneration markers, and metabolic and homeostatic regulators, reflecting the variety of biological processes involved in the dystrophic state.

### 3.2. Upregulated Genes Reveal Amplified Inflammatory, Interferon-Associated, and Innate-Sensing Programs in Dystrophic Soleus

Having established that δ-sarcoglycan deficiency produces a broadly shared but not identical transcriptional response in soleus and EDL, we next focused on genes upregulated in *Sgcd*^−/−^ muscles to identify the biological programs represented by this component of the dystrophic transcriptome. GO and KEGG enrichment analyses of upregulated genes from each muscle revealed immune and inflammatory terms in both dystrophic muscles, including innate immune response, immune response-regulating signaling pathways, cytokine-mediated signaling, leukocyte migration, myeloid leukocyte activation, chemokine signaling, toll-like receptor signaling, NF-κB signaling, and phagosome-related pathways (Figure 2A,B; Supplementary Table S2). Thus, the upregulated components of the dystrophic response were not exclusive to soleus; both muscles showed related immune programs in the absence of δ-sarcoglycan.

To resolve the gene-level organization of this response, we analyzed curated inflammatory and innate-sensing gene modules. A focused heatmap showed strong induction of genes associated with NF-κB signaling, NOD-like receptor signaling, TLR signaling, RLR-mediated RNA sensing, and downstream type-I interferon-stimulated genes in *Sgcd*^−/−^ muscles (Figure 2C). Within these modules, soleus displayed stronger induction of several inflammatory and innate immune-associated transcripts, including cytokine and chemokine genes, TLR/NF-κB pathway components, interferon-associated genes, and selected immune-cell-associated markers such as *Cd86*, *Ccl2*, *Ccl7*, *Ccl8*, *Cxcl10*, *Myd88*, *Tlr2*, *Tlr7*, *Irf7*, *Ifih1*, *Stat1*, *Stat2*, *Isg15*, and *Mx1*. Comparative GO enrichment between dystrophic soleus and EDL further showed stronger enrichment in soleus for leukocyte migration, regulation of innate immune response, cytokine-mediated signaling pathway, myeloid leukocyte activation, response to viruses, defense response to viruses, and regulation of inflammatory response (Figure 2D).

We next asked whether the inflammatory programs enriched in dystrophic soleus were accompanied by greater gene-level transcriptional changes than those observed in EDL. We examined the log2 fold-change distributions (*Sgcd*^−/−^ versus WT) for TLR, NOD-like receptor, NF-κB, RLR/IFN-I, and ISG-associated gene sets. These distributions were significantly more positive in soleus than in EDL (paired Wilcoxon signed-rank test; Figure 2E), indicating stronger induction of these inflammatory and innate-sensing programs in the soleus. RT-qPCR analysis confirmed significantly higher expression of *Tlr2* and *Tlr4* in dystrophic soleus relative to EDL, with *Myd88*, *Nfkb2*, *Irf7*, and *Cd68* showing higher expression in the same direction (Figure 2F). Additional comparisons against wild-type controls confirmed upregulation of TLR/NF-κB and interferon-stimulated genes in both dystrophic muscles (Supplementary Figure S2). Together, the RNA-seq analysis and RT-qPCR results support the conclusion that dystrophic soleus amplifies selected inflammatory, interferon-associated, and innate-sensing transcriptional features within a broadly shared dystrophic response.

### 3.3. Downregulated Genes Reveal Suppression of Metabolic and Mitochondrial Transcriptional Programs in Dystrophic Muscles

Having characterized the upregulated inflammatory and immune-associated programs in *Sgcd*^−/−^ muscles, we next examined downregulated genes to define the transcriptional programs suppressed in the dystrophic state. GO and KEGG enrichment analyses of downregulated genes in both muscles identified metabolic processes related to generation of precursor metabolites and energy, ribose phosphate and ribonucleotide metabolism, purine ribonucleotide metabolism, fatty acid metabolic processes, small-molecule catabolism, organic acid catabolism, carboxylic acid catabolism, cellular respiration, oxidative phosphorylation, carbon metabolism, and peroxisome pathways (Figure 3A,B; Supplementary Table S3). These findings indicate that δ-sarcoglycan deficiency suppresses coordinated metabolic gene programs in both oxidative and glycolytic muscles.

A focused heatmap of metabolic genes revealed coordinated repression of transcripts associated with BCAA metabolism, mitochondrial pathways, the TCA cycle, fatty acid oxidation, and oxidative phosphorylation in *Sgcd*^−/−^ muscles, including *Ppargc1a*, *Opa1*, *Ogdh*, *Cpt2*, *Acadl*, *Ndufs3*, and *Atp5b* (Figure 3C). Comparative enrichment analysis further showed that downregulated genes from both dystrophic soleus and EDL were significantly associated with metabolic and mitochondrial processes (Figure 3D). Notably, dystrophic soleus displayed prominent enrichment of downregulated pathways related to fatty acid oxidation, lipid catabolism, fatty acid beta-oxidation, acyl-CoA metabolism, aerobic respiration, and oxidative phosphorylation. This pattern suggests that δ-sarcoglycan deficiency strongly suppresses oxidative and lipid-based metabolic programs in soleus, a muscle normally reliant on mitochondrial energy metabolism.

To determine whether the pathway-level findings reflected coordinated repression across individual genes, we examined the log2 fold-change distributions (*Sgcd*^−/−^ versus WT) for oxidative phosphorylation, fatty acid oxidation, TCA cycle, BCAA metabolism, and mitochondrial gene sets. These distributions were consistently negative in both muscles, confirming coordinated repression in the dystrophic state, and were significantly more negative in soleus than in EDL (paired Wilcoxon signed-rank test; Figure 3E), indicating stronger suppression of oxidative and mitochondrial programs in soleus. Together, these analyses establish broad repression of oxidative and mitochondrial transcriptional programs as a major component of the δ-sarcoglycan-deficient muscle transcriptome, with the soleus more strongly affected.

### 3.4. Computational Deconvolution Supports Macrophage-Associated Remodeling in δ-Sarcoglycan-Deficient Muscle

Because inflammatory and immune-cell-associated transcripts were increased in dystrophic muscles, we used computational deconvolution to estimate immune-cell-associated signatures from bulk RNA-seq profiles. quanTIseq analysis indicated genotype-associated changes in immune-cell fractions across soleus and EDL (Figure 4A). Among the inferred populations, M1 macrophage estimates were significantly increased in both *Sgcd*^−/−^ soleus and EDL relative to their corresponding WT controls (Figure 4B). Notably, where WT soleus showed lower baseline M1 estimates than WT EDL, dystrophic soleus exhibited a pronounced increase relative to this low baseline, approaching the levels observed in dystrophic EDL. Although the direct comparison between dystrophic muscles did not reach statistical significance, this pattern indicates a marked relative expansion of the M1-associated signature in the soleus. In parallel, mMCP-counter also detected genotype-associated shifts in immune and stromal cell-associated signatures, including changes in monocyte/macrophage-associated scores (Figure 4C). Notably, monocyte/macrophage estimates from mMCP-counter correlated with quanTIseq macrophage estimates across samples (Spearman rho = 0.762, *p* = 0.0059; Figure 4D). The convergence of two deconvolution approaches supports a macrophage-associated component in the dystrophic transcriptional state. This macrophage-associated signature is consistent with the enrichment of TLR/NF-κB and interferon-related pathways observed in dystrophic muscle, suggesting that infiltrating or expanding M1 macrophages may contribute to the inflammatory transcriptional state. However, because these analyses are derived from bulk RNA-seq, they should be interpreted as inferred cell-associated signatures and require validation by flow cytometry or spatial profiling.

### 3.5. (-)-Epicatechin Exerts Context-Dependent Transcriptional Effects and Attenuates the Inflammatory/Innate-Sensing Module in Dystrophic Soleus

We next evaluated whether (-)-epicatechin modulates the disease-associated transcriptional state. The distribution of log_2_ fold changes between (-)-epicatechin-treated and vehicle-treated samples revealed a muscle- and genotype-dependent response. In *Sgcd*^−/−^ soleus, treatment induced 38 upregulated and 101 downregulated genes, whereas *Sgcd*^−/−^ EDL showed a more limited response, with 20 upregulated and 39 downregulated genes. Among the downregulated transcripts, *Mx1* was shared by both muscles, while *Oas1a* and *Ifit2* were identified in soleus and *Ifit1* and *Ifit3* in EDL. WT muscles also responded to (-)-epicatechin, but the transcriptional profile was distinct: WT soleus showed 101 upregulated and 158 downregulated genes, whereas WT EDL showed 272 upregulated and 199 downregulated genes (Figure 5A; Supplementary Table S4). At the global transcriptomic level, principal component analysis showed that (-)-epicatechin-treated *Sgcd*^−/−^ samples remained closer to their corresponding vehicle-treated *Sgcd*^−/−^ groups than to WT samples (Supplementary Figure S3; Supplementary Table S6). Thus, treatment did not broadly reverse the genotype-driven transcriptional separation. Moreover, the sets of genes responsive to (-)-epicatechin showed limited overlap across genotypes and muscles, further supporting a context-dependent transcriptional response. GO enrichment analysis revealed that (-)-epicatechin modulated distinct biological processes depending on genotype. In *Sgcd*^−/−^ muscle, the strongest enrichment was observed among downregulated genes involved in processes related to antiviral, interferon-related, and innate immune pathways, including viral defense programs, interferon-β-associated signaling, regulation of innate immunity, and negative regulation of viral processes (Figure 5C). In WT muscles, by contrast, the most prominent enrichment was observed among upregulated genes, which were associated with muscle and developmental processes, including muscle cell differentiation, muscle organ development, actomyosin structure organization, myofibril assembly, extracellular matrix organization, and striated muscle contraction (Figure 5C). Complete enrichment results for up- and downregulated genes across muscles and genotypes are provided in Supplementary Table S4.

A focused heatmap further showed that (-)-epicatechin reduced expression of TLR/NF-κB, RLR-mediated RNA sensing, and downstream type-I interferon-stimulated genes in *Sgcd*^−/−^ soleus (Figure 5B). This reduction encompassed innate immune receptors and adaptors, including *Tlr4*, *Tlr2*, *Myd88*; regulatory and RNA-sensing components, including *Nfkb2*, *Irf7*, *Rigi*, *Lgp2*, and *Ifih1*; and downstream chemokine and interferon-stimulated genes, including *Ifit3*, *Ifit1*, *Ddx60*, *Mx1*, *Isg15*, and several Oas-family members such as *Oas3*, *Oasl2*, *Oasl1*, and *Oas1a*. RT-qPCR validation confirmed a significant reduction in *Tlr2*, *Cxcl9*, *Nfkb2*, *Irf7*, *Rigi*, *Ifit1*, *Mx1*, *Isg15*, *Oas3*, and *Oasl2* after treatment (Figure 5D). Together, these results indicate that (-)-epicatechin does not produce a uniform anti-inflammatory transcriptional response across muscles and genotypes. Instead, its most prominent effect in dystrophic soleus was the coordinated attenuation of the same interferon/innate-sensing module that characterized the dystrophic response, spanning TLR/NF-κB signaling, RLR-mediated RNA sensing, and downstream interferon-stimulated genes (Figure 2). This overlap between the disease-associated module and the epicatechin-responsive genes points to the interferon/innate-sensing axis as the main transcriptional target of the treatment in dystrophic soleus.

## 4. Discussion

This study provides a focused transcriptomic analysis of δ-sarcoglycan deficiency using two functionally distinct skeletal muscles, soleus and EDL, and evaluates the transcriptional effects of (-)-epicatechin in this disease context. The central finding is that interferon-associated inflammatory remodeling and oxidative metabolic repression define the skeletal muscle transcriptomic landscape of δ-sarcoglycan deficiency. Within this shared state, soleus displays stronger inflammatory, innate-sensing, and inferred macrophage-associated transcriptional features. In addition, (-)-epicatechin most prominently attenuates an interferon/innate-sensing transcriptional module in dystrophic soleus, as supported by both RNA-seq and RT-qPCR validation.

A key implication of these results is that the dystrophic inflammatory response is not exclusive to one muscle type. Both soleus and EDL showed enrichment of interferon-associated, inflammatory and TLR/NF-κB pathways. However, the magnitude and cellular context of this response differed between muscles. Soleus showed stronger enrichment of leukocyte migration, innate immune response regulation, cytokine signaling, myeloid leukocyte activation, and antiviral/interferon-associated processes. This distinction is important because it reframes the muscle-specific component of LGMD2F not as the presence or absence of inflammation, but as differential amplification of selected inflammatory and immune-remodeling programs within a shared disease state. The cellular basis of this stronger soleus signature warrants caution. Deconvolution did not indicate a greater absolute immune-cell content in dystrophic soleus than in EDL: although M1-macrophage estimates increased with disease in the soleus, they only approached—rather than exceeded—those of the EDL. The amplified inflammatory and interferon enrichment in the soleus is therefore unlikely to be explained simply by a higher number of infiltrating immune cells, and may instead reflect, at least in part, a cell-intrinsic response of oxidative myofibers and other resident cells to dystrophic injury.

The inflammatory program identified here included genes associated with TLR/NF-κB signaling, NOD-like receptor signaling, RLR-mediated RNA sensing, and downstream type-I interferon-stimulated genes. These patterns are consistent with chronic sterile injury, nucleic-acid sensing, and immune activation in dystrophic muscle and align with prior evidence implicating NF-κB, TLR signaling, and inflammasome-related mechanisms in muscle degeneration and inflammatory muscle remodeling [8,30,31,32]. The activation of RLR-mediated RNA sensing in a non-infectious, genetic disease is consistent with the concept that endogenous (self) RNA released from damaged myofibers can engage these sensors, as described in sterile inflammation and in myotonic dystrophy [10,13]. In this respect, the enrichment of antiviral and interferon-related terms does not imply viral infection; rather, it reflects the engagement of the same RNA-sensing and interferon machinery by endogenous stimuli in the dystrophic muscle. Together, these findings highlight TLR/NF-κB, RLR-mediated RNA sensing, and type-I interferon-stimulated programs as prominent transcriptional modules within the LGMD2F transcriptome.

At the gene level, this inflammatory signature was organized across several complementary innate-immune components. Increased expression of *Tlr2*, *Tlr*4, and *Myd88* was consistent with enhanced TLR-associated inflammatory signaling, whereas *Rigi*, *Ifih1*, *Lgp2*, *Irf7*, *Stat1*, *Stat2* and downstream genes such as *Isg15*, *Mx1*, and Ifit-family members defined a coordinated RNA-sensing/interferon-associated transcriptional program. In parallel, increased expression of chemokines including *Ccl2*, *Ccl7*, *Ccl8*, and *Cxcl10* provides a transcriptional link to the myeloid-associated remodeling inferred by deconvolution. Persistent overexpression of *Ccl2*, *Ccl7*, and *Ccl8* has also been reported in mdx skeletal muscle and associated with macrophage and T-cell recruitment [33,34]. Similar enrichment of innate immune, macrophage-associated, and pro-inflammatory chemokine programs has recently been reported in alpha-sarcoglycanopathy [9], suggesting that inflammatory remodeling may represent a broader feature of sarcoglycan-deficient muscle rather than an isolated consequence of δ-sarcoglycan loss.

Because the present study primarily measures mRNA abundance, future protein-level and spatial analyses of NF-κB, STAT/IRF, and RLR-mediated RNA-sensing activity will be valuable for defining the cellular sources and mechanistic consequences of this module.

In parallel with inflammatory remodeling, δ-sarcoglycan deficiency was associated with repression of oxidative and mitochondrial transcriptional programs. Downregulated pathways included oxidative phosphorylation, fatty acid oxidation, TCA cycle, BCAA metabolism, mitochondrial translation, and related energy-metabolic processes. This suggests that inflammatory/interferon remodeling and metabolic suppression are co-occurring features of the dystrophic state. In soleus, a muscle normally enriched for oxidative metabolism, repression of these programs may be particularly relevant to disease vulnerability and tissue remodeling.

Notably, the co-occurrence of mitochondrial repression and RLR-mediated RNA sensing suggests that mitochondrial dysfunction contributes to the innate-sensing response. Under mitochondrial stress, mitochondrial-encoded RNA can escape into the cytosol and engage the RIG-I-like receptors RIG-I and MDA5—both upregulated in our dataset—triggering MAVS-dependent signaling and a type-I interferon response [35,36]. Consistent with this, disrupted mitochondrial dynamics have been shown to promote BAX-dependent release of mitochondrial RNA that activates RIG-I–MAVS signaling [37]. However, in our dataset, BAX was not upregulated in the dystrophic muscles, and mtDNA-encoded transcripts were not detected as differentially expressed in our analyses. Together, these mechanisms provide a plausible route by which the mitochondrial repression observed here could feed into the interferon program in dystrophic soleus. In our own dataset, the repression of nuclear-encoded mitochondrial programs and the induction of interferon-stimulated genes were co-regulated across samples, with the RNA-sensing (RLR) arm appearing to predominate over the cytosolic DNA-sensing (cGAS–STING) arm—observations that are consistent with, but do not prove, a contribution of mitochondrial RNA sensing to the interferon signature and warrant dedicated investigation.

Computational deconvolution also supported a macrophage-associated component of the dystrophic transcriptome in both muscles. Both quanTIseq and mMCP-counter detected genotype-associated changes in immune-cell-associated signatures, and monocyte/macrophage-related estimates correlated across methods. Importantly, these estimates did not indicate a greater immune-cell content in soleus than in EDL; rather, they point to a macrophage-associated component of the shared dystrophic response that coexists with the cell-intrinsic contribution noted above. These results remain an inference from bulk transcriptomic data and are interpreted here as supporting rather than quantitative evidence.

The (-)-epicatechin results add a perturbational dimension to the study. Treatment effects were strongly context-dependent: WT muscles showed transcriptional changes related to muscle and developmental processes, whereas *Sgcd*^−/−^ soleus showed coordinated downregulation of inflammatory, antiviral, interferon-stimulated, and innate-sensing genes. This suggests that (-)-epicatechin does not simply act as a broad anti-inflammatory compound but modulates a disease-associated transcriptional module that is most evident in the dystrophic soleus context. Importantly, these data support transcriptional attenuation of inflammatory/innate-sensing signatures, not therapeutic efficacy by themselves. Nevertheless, recent evidence in the same *Sgcd*^−/−^ model indicates that the effects of (-)-epicatechin are not restricted to transcriptional modulation. (-)-Epicatechin treatment has been reported to reduce fibrosis in dystrophic triceps and gastrocnemius, increase myofiber cross-sectional area in triceps, and alter myogenic regulators in a muscle-dependent manner [38]. These findings provide complementary evidence that (-)-epicatechin can induce structural and myogenic changes in dystrophic skeletal muscle, while also reinforcing the notion that its effects vary considerably among muscle groups. Functional studies will still be required to determine whether the transcriptional changes observed here translate into improved muscle pathology or performance. The predominance of this effect in soleus may reflect the larger dynamic range available in the muscle where the inflammatory/interferon module is most strongly amplified, although muscle-type-specific pharmacodynamics and bioavailability were not assessed and cannot be excluded.

Several limitations should be considered. First, the study uses bulk RNA-seq from pooled muscle samples, with three independent pools per condition. Pooling was necessary to obtain sufficient RNA input from these small muscles and, while it preserves biological replication at the level of the experimental unit, it averages inter-individual variability within each replicate and may influence the number and magnitude of detected differentially expressed genes. To mitigate this, key differentially expressed genes were independently validated by RT-qPCR. Nonetheless, studies using individual biological replicates would be valuable to refine these estimates. Second, because our analysis is based on bulk RNA-seq, it cannot resolve whether the inflammatory and interferon signatures arise from an increased proportion of infiltrating immune cells or from an intrinsic response of muscle fibers to damage; both sources are likely to contribute, as RLRs, TLRs, and interferon-stimulated genes are expressed by myeloid cells but also by muscle fibers and other resident cell types. Although deconvolution suggested a macrophage-associated component, this approach has important limitations in skeletal muscle: the methods used (quanTIseq and mMCP-counter) were developed and validated primarily for blood and tumor contexts, and their performance in a myofiber-dominated tissue has not been formally established. The moderate concordance between methods (Spearman rho = 0.762) and the modest absolute changes in estimated cell fractions further warrant cautious interpretation, and non-immune resident cells such as fibroblasts may contribute substantially to the bulk inflammatory signal. We therefore present the deconvolution results as a supporting, inferential line of evidence rather than a definitive quantification of immune-cell content, and single-cell or spatial transcriptomics together with cellular validation (e.g., immunostaining or flow cytometry) will be required to attribute these signatures to specific cell populations. Third, pathway analyses identify coordinated transcriptional signatures but do not establish pathway activity at the protein or functional level. Despite these limitations, the integration of differential expression, pathway analysis, immune deconvolution, and qPCR validation provides a coherent transcriptomic framework for understanding immune-metabolic remodeling in δ-sarcoglycan deficiency. Finally, our analysis represents a single disease time point (8–12 weeks), and (-)-epicatechin was administered within this same window, so the transcriptional response reported here corresponds to treatment initiated at an established disease stage. Although the natural history of this model indicates that muscle pathology is already established and relatively stable at this age [5], a cross-sectional design cannot formally distinguish transcriptional changes that are primary to the disease from those secondary to progressive muscle wasting, nor can it resolve the temporal sequence of inflammatory and metabolic remodeling. Longitudinal studies, and comparisons of early versus late treatment initiation, would help address these questions and clarify how disease stage shapes both the dystrophic transcriptome and its response to (-)-epicatechin.

Overall, our findings support a model in which δ-sarcoglycan deficiency induces a shared interferon-associated inflammatory and metabolic dystrophic state across skeletal muscles, with dystrophic soleus showing stronger innate-sensing, inflammatory and macrophage-associated transcriptional features. The partial attenuation of this module by (-)-epicatechin in dystrophic soleus suggests the interferon/innate-sensing axis as a modifiable transcriptional component of LGMD2F muscle pathology.

## 5. Conclusions

In summary, interferon-associated inflammatory remodeling and oxidative metabolic repression define the skeletal muscle transcriptomic landscape of δ-sarcoglycan deficiency. Within this shared dystrophic state, soleus displays stronger innate-sensing and inflammatory features, together with inferred macrophage-associated signatures. (-)-Epicatechin partially attenuates a defined inflammatory/interferon-stimulated and innate-sensing transcriptional module in dystrophic soleus, suggesting this axis as a modifiable component of LGMD2F-associated muscle remodeling.

## Figures and Tables

**Figure 1:**
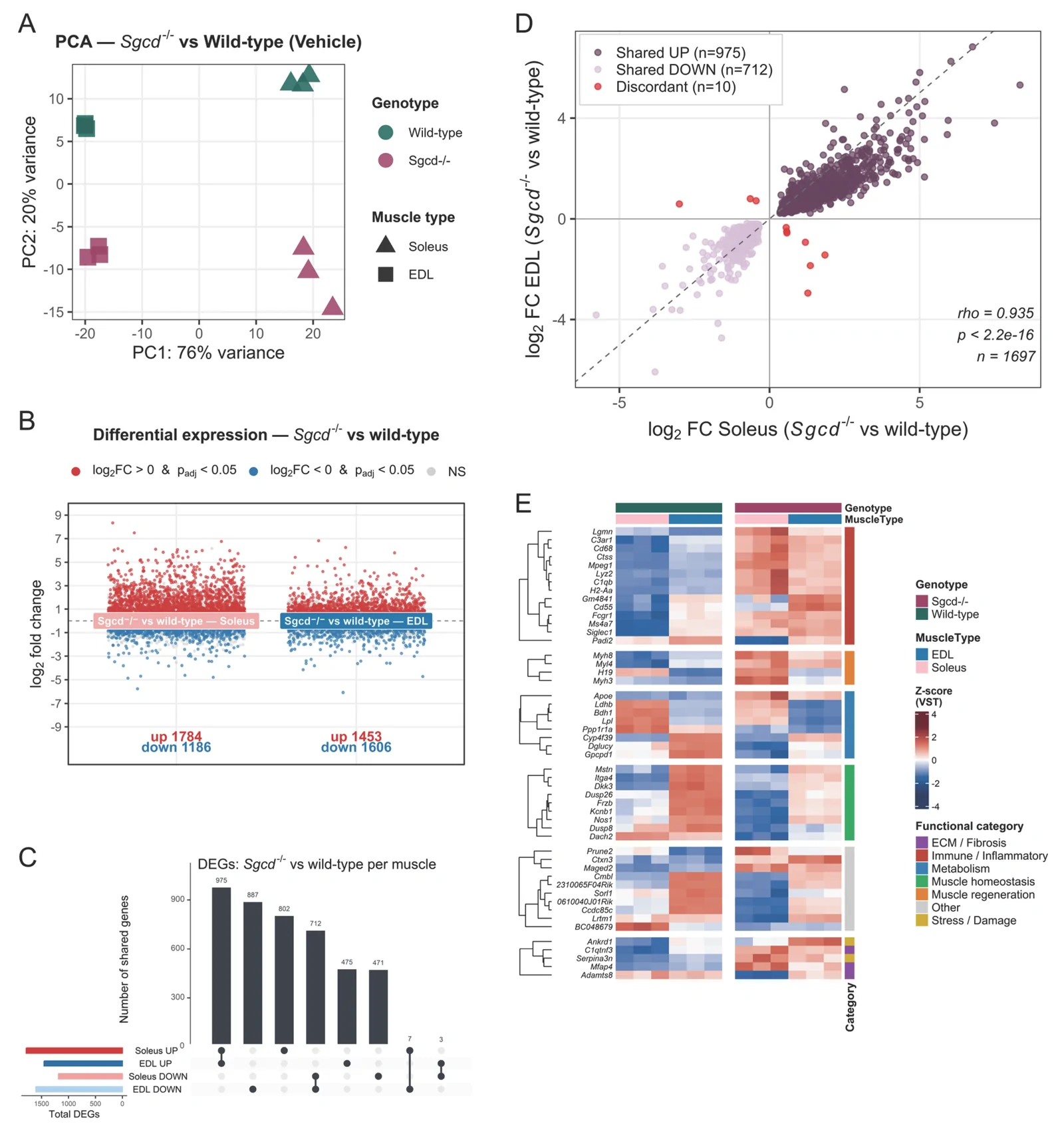
Global transcriptomic profiling reveals shared dystrophic remodeling and preserved muscle-type identity in δ-sarcoglycan deficiency. (**A**) Principal component analysis (PCA) of bulk RNA-seq samples from WT and *Sgcd*^−/−^ soleus and EDL muscles. Samples are colored by genotype and shaped by muscle type. PC1 separates muscle identity, whereas PC2 captures genotype-associated variation. (**B**) Differential expression overview for *Sgcd*^−/−^ versus WT comparisons in soleus and EDL. Red points indicate significantly upregulated genes, and blue points indicate significantly downregulated genes. (**C**) UpSet plot summarizing the overlap of upregulated and downregulated genes between dystrophic soleus and EDL. (**D**) Correlation of log2 fold-change values for shared differentially expressed genes between soleus and EDL, showing strong concordance of the dystrophic response while identifying genes with larger effect sizes in one muscle. Correlation was assessed using Spearman’s rank correlation (rho = 0.935, *p* < 2.2 × 10^−16^). (**E**) Heatmap of representative disease-associated genes across WT and *Sgcd*^−/−^ soleus and EDL muscles. Values represent row-scaled variance-stabilized expression values.

**Figure 2:**
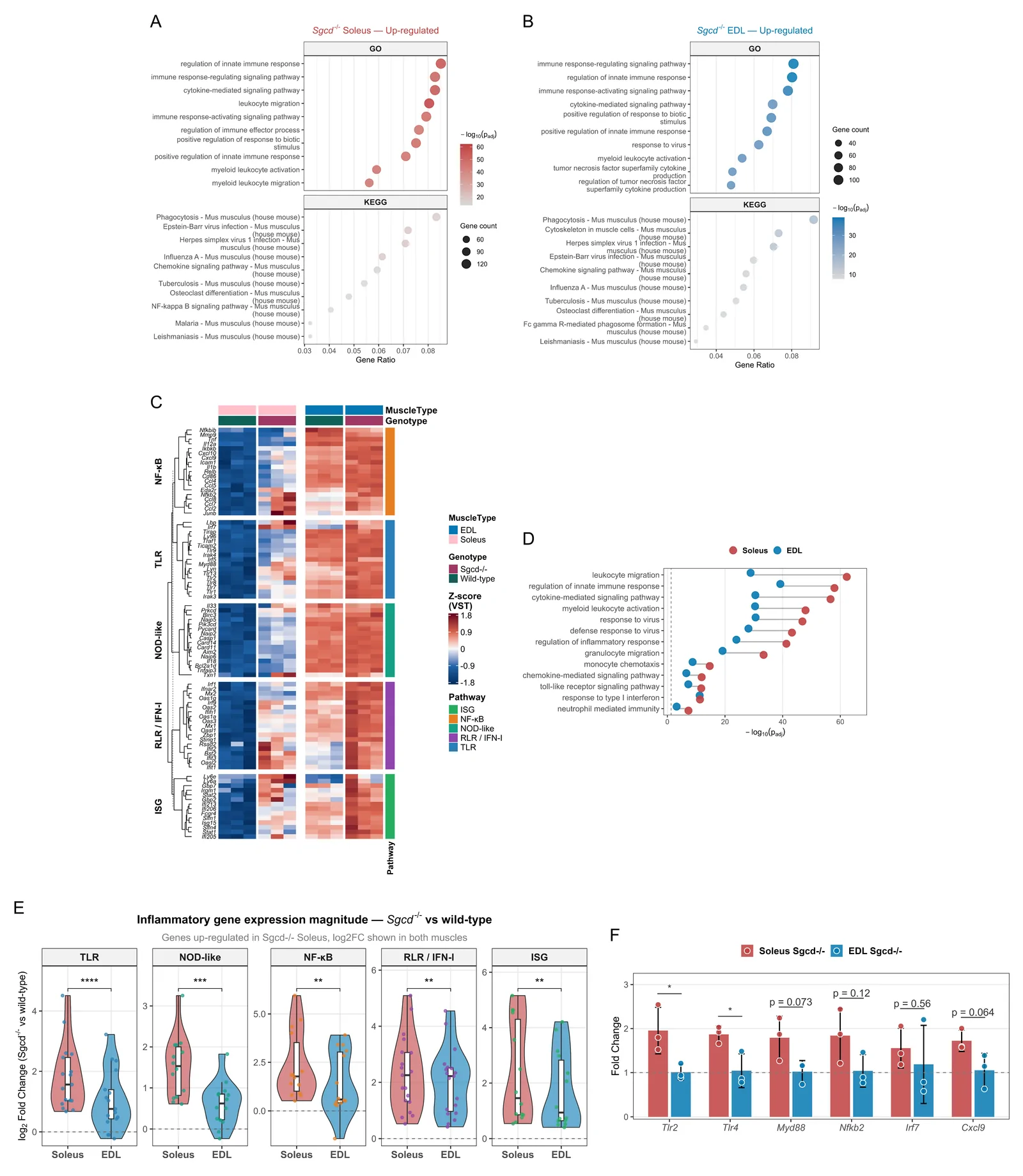
Upregulated genes reveal amplified inflammatory, interferon-associated, and innate-sensing programs in dystrophic soleus. (**A**,**B**) GO and KEGG enrichment analyses of upregulated genes in *Sgcd*^−/−^ soleus and *Sgcd*^−/−^ EDL muscles. (**C**) Focused heatmap of genes associated with NF-κB signaling, NOD-like receptor signaling, TLR signaling, RLR-mediated RNA sensing, and downstream type-I interferon-stimulated genes. Values represent row-scaled variance-stabilized expression values. (**D**) Comparative inflammatory GO enrichment between dystrophic soleus and EDL. (**E**) Violin plots showing log2 fold-change (*Sgcd^−/−^* versus WT) for TLR, NOD-like, NF-κB, RLR/IFN-I, and ISG-associated gene sets. Each point represents one gene; statistical comparison between soleus and EDL was performed using a paired Wilcoxon signed-rank test (** *p* < 0.01; *** *p* < 0.001; **** *p* < 0.0001). (**F**) RT-qPCR validation of selected inflammatory and innate-sensing genes in *Sgcd^−/−^* soleus and EDL muscles. Expression was normalized to Tbp and is shown as fold change; bars represent mean ± SD (*n* = 3 per group). Statistical comparisons were performed using two-tailed Student’s *t*-tests (* *p* < 0.05).

**Figure 3:**
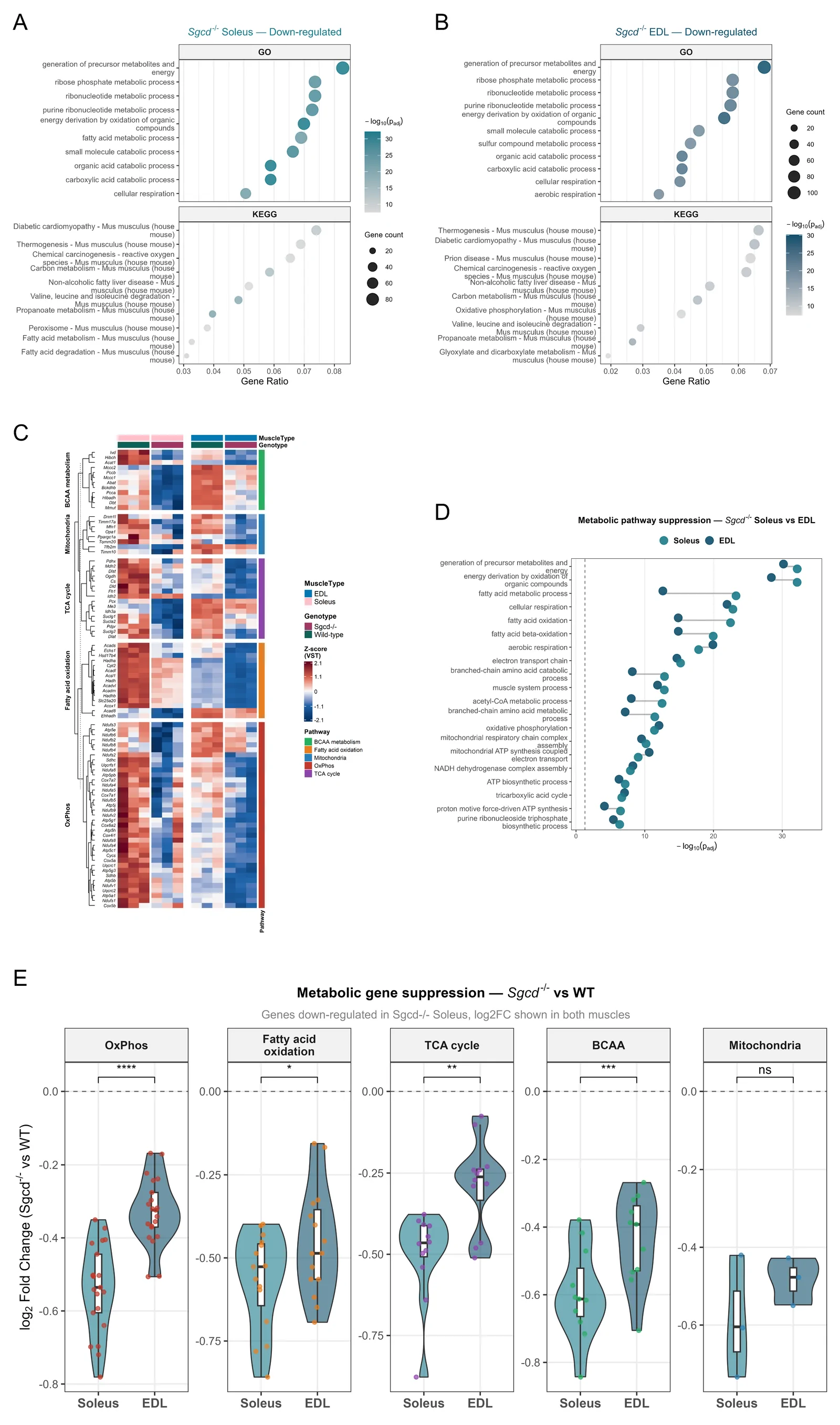
Downregulated genes reveal suppression of metabolic and mitochondrial transcriptional programs in dystrophic muscles. (**A**,**B**) GO and KEGG enrichment analyses of downregulated genes in Sgcd^−/−^ soleus and Sgcd^−/−^ EDL muscles. (**C**) Heatmap of genes associated with BCAA metabolism, mitochondrial pathways, TCA cycle, fatty acid oxidation, and oxidative phosphorylation. Genes were selected from curated gene sets for these metabolic pathways and restricted to those significantly downregulated (adjusted *p* < 0.05) in at least one dystrophic muscle. Values represent row-scaled VST-normalized expression. (**D**) Comparative metabolic pathway suppression between dystrophic soleus and EDL. (**E**) Violin plots showing log2 fold-change (Sgcd^−/−^ versus WT) for OxPhos, fatty acid oxidation, TCA cycle, BCAA metabolism, and mitochondrial gene sets. Each point represents one gene; statistical comparison between soleus and EDL was performed using a paired Wilcoxon signed-rank test (ns, not significant; * *p* < 0.05; ** *p* < 0.01; *** *p* < 0.001; **** *p* < 0.0001).

**Figure 4:**
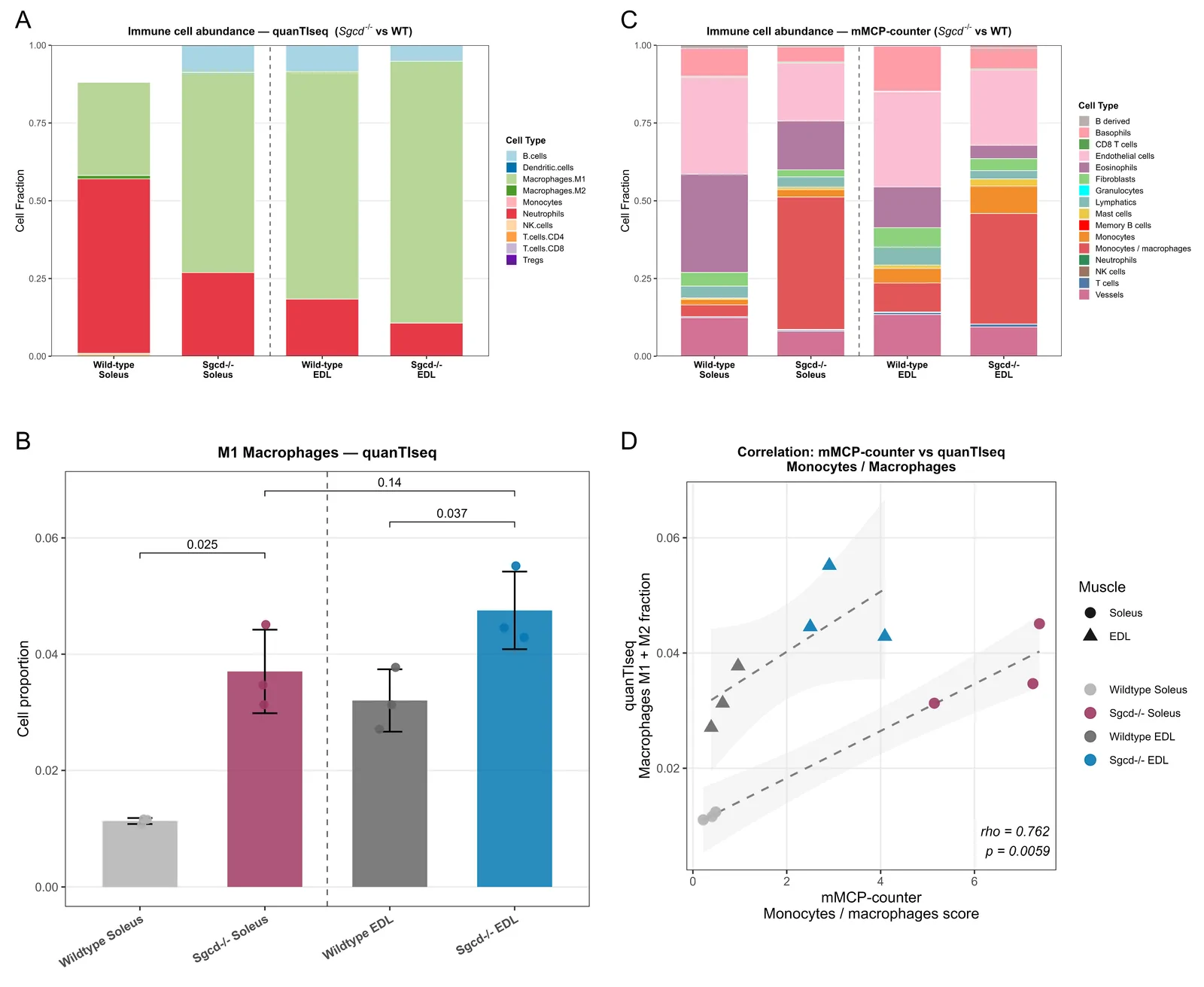
Computational deconvolution supports macrophage-associated remodeling in *Sgcd^−/−^* muscle. (**A**) quanTIseq-estimated immune-cell fractions across WT and *Sgcd^−/−^* soleus and EDL muscles. (**B**) Estimated M1 macrophage proportions by quanTIseq. Bars represent mean ± SD (*n* = 3 per group); statistical comparisons were performed using two-tailed Student’s *t*-tests, with exact *p*-values shown. (**C**) mMCP-counter-estimated immune and stromal cell-associated signatures across groups. (**D**) Correlation between mMCP-counter monocyte/macrophage scores and quanTIseq macrophage estimates across samples, assessed using Spearman’s rank correlation (rho = 0.762, *p* = 0.0059).

**Figure 5:**
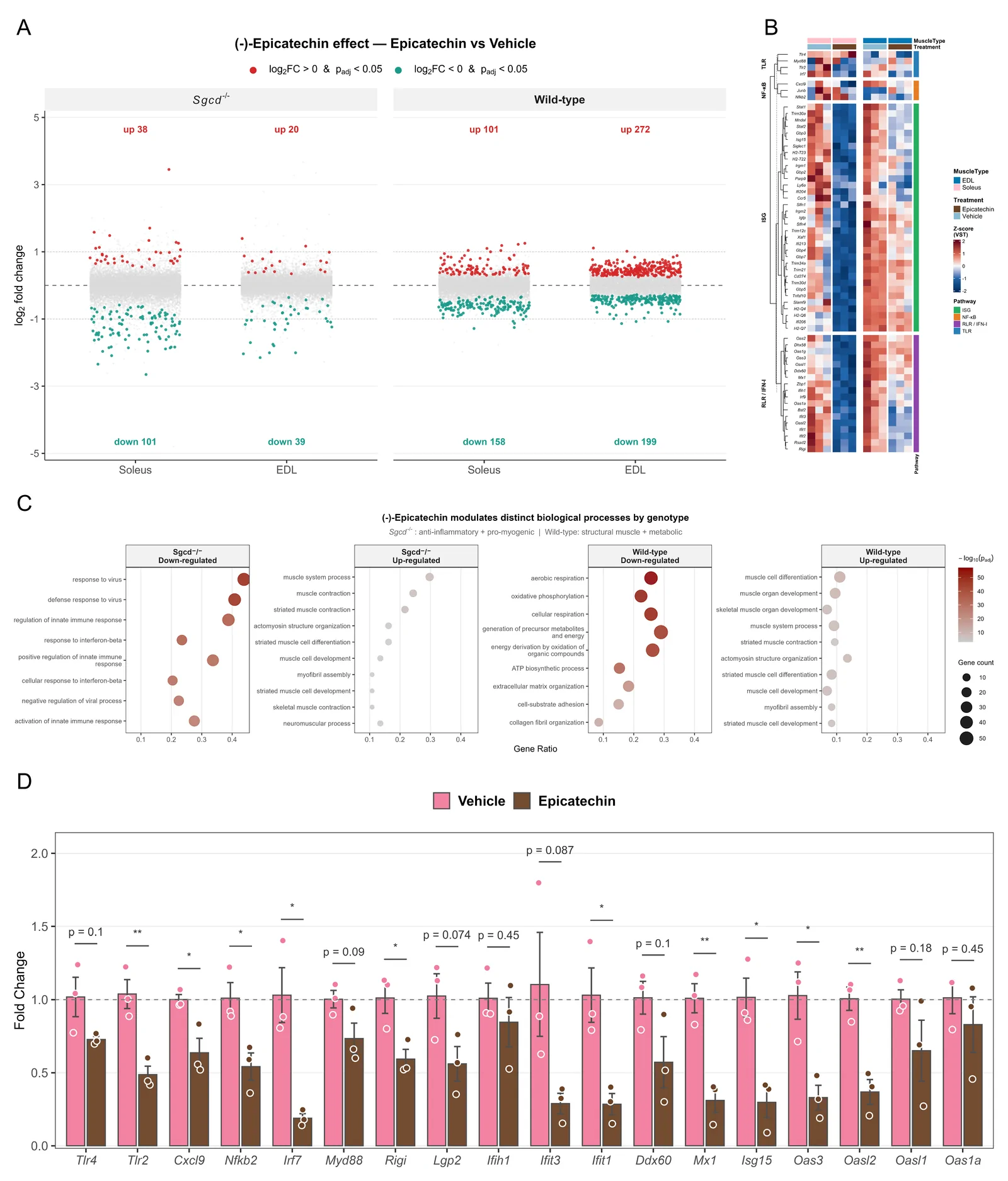
(-)-Epicatechin attenuates an inflammatory/innate-sensing transcriptional module in dystrophic soleus. (**A**) Distribution of log_2_ fold changes for differential expression after (-)-epicatechin treatment relative to vehicle in *Sgcd*^−/−^ soleus, *Sgcd*^−/−^ EDL, WT soleus, and WT EDL. Each point represents one gene; colored points indicate differentially expressed genes (adjusted *p* < 0.05, DESeq2 with Benjamini–Hochberg correction). (**B**) Focused heatmap of TLR/NF-κB, RLR-mediated RNA sensing, and downstream type-I interferon-stimulated genes after treatment. Values represent row-scaled VST-normalized expression. (**C**) GO enrichment analysis showing that (-)-epicatechin suppresses inflammatory and antiviral processes in *Sgcd*^−/−^ muscle while modulating muscle and developmental processes in WT muscle. (**D**) RT-qPCR validation of selected inflammatory and interferon-associated genes in *Sgcd*^−/−^ soleus treated with vehicle or (-)-epicatechin. Expression was normalized to Tbp and is shown as fold change relative to vehicle; bars represent mean ± SD (*n* = 3 per group). Statistical comparisons were performed using two-tailed Student’s *t*-tests. Significance is indicated by asterisks (* *p* < 0.05; ** *p* < 0.01), and exact *p*-values are shown for non-significant comparisons.

## Data Availability

The raw sequencing data generated in this study have been deposited in the Gene Expression Omnibus (GEO) under accession number GSE327571.

## Supplementary Materials

The following supporting information can be downloaded at https://www.mdpi.com/article/10.3390/biom16101511/s1. Figure S1: Fiber-type-specific transcriptional identity is preserved in *Sgcd^−/−^* muscle. Figure S2: RT-qPCR validation of the inflammatory transcriptional signature in *Sgcd^−/−^* muscle. Figure S3: (-)-Epicatechin exerts genotype- and muscle-specific transcriptional effects. Table S1: Differentially expressed genes (*Sgcd^−/−^* vs. WT), related to Figure 1. Table S2: DEGs and GO/KEGG enrichment, related to Figure 2. Table S3: DEGs and GO/KEGG enrichment, related to Figure 3. Table S4: DEGs and GO enrichment of epicatechin-modulated genes, related to Figure 5. Table S5: DEGs and GO/KEGG enrichment, related to Figure S1. Table S6: GO enrichment of epicatechin-modulated genes, related to Figure S3.
