# Supplementary material for: Interferon-Associated Inflammatory Remodeling and Oxidative Metabolic Repression Define the Skeletal Muscle Transcriptomic Landscape of δ-Sarcoglycan Deficiency
Source: Biomolecules. 2026 Oct 17;16(10):1511. doi: 10.3390/biom16101511 (PMC13702233; doi:10.3390/biom16101511)
Supplement: Supplementary file 1 [file biomolecules-16-01511-s001.zip › Supplementary figures_/Supplementary figure S1.pdf]

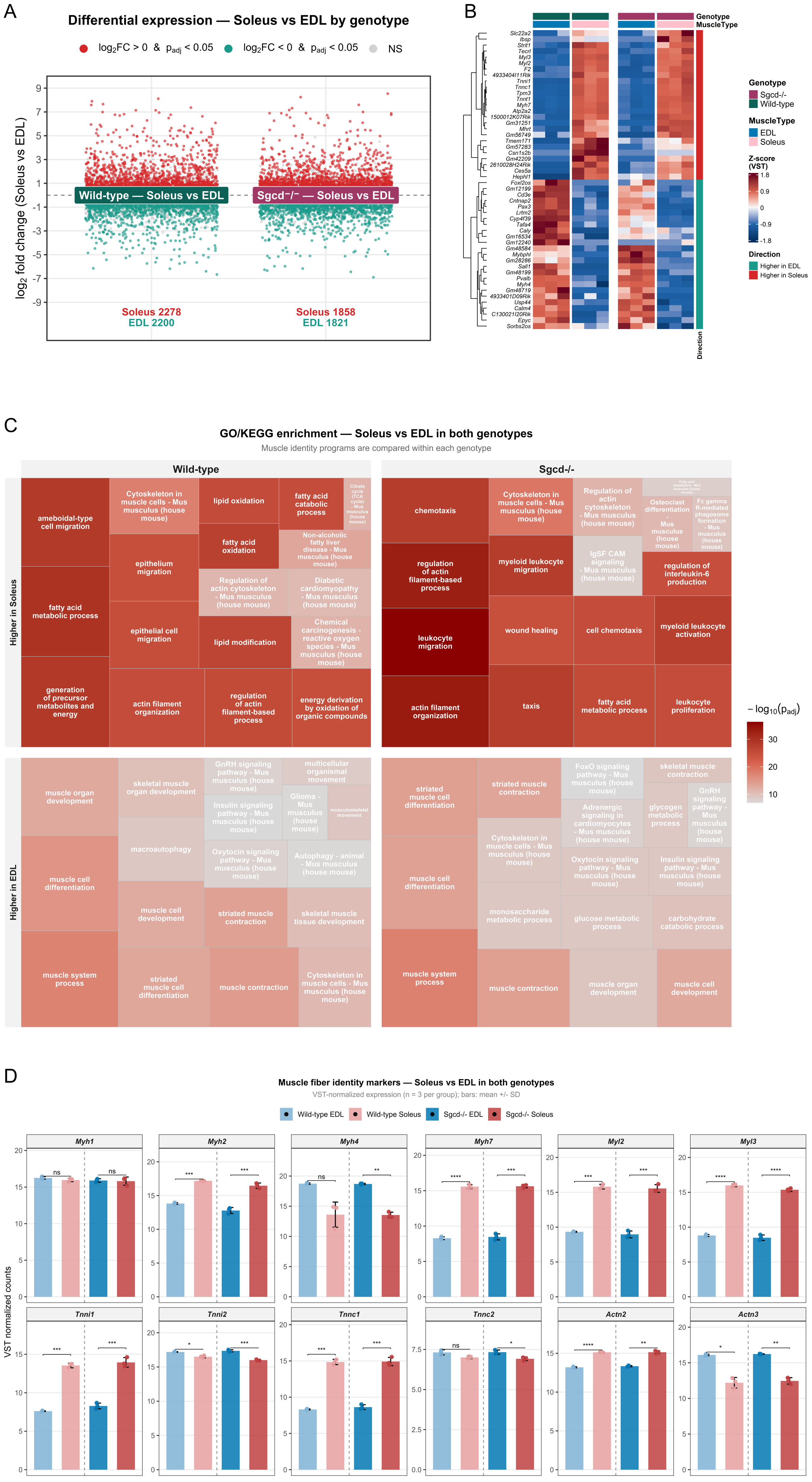

A
Differential expression — Soleus vs EDL by genotype
log2FC > 0 & padj < 0.05
log2FC < 0 & padj < 0.05
NS
log2 fold change (Soleus vs EDL)
Wild-type — Soleus vs EDL
Sgcd−/− — Soleus vs EDL
Soleus 2278
EDL 2200
Soleus 1858
EDL 1821
B
Genotype
MuscleType
Slc22a2
Ibsp
Strit1
Tecrl
Myl3
Myl2
F2
4933404I11Rik
Tnni1
Tnnc1
Tpm3
Tnnt1
Myh7
Atp2a2
1500012K07Rik
Gm31251
Mhrt
Gm56749
Tmem171
Gm57283
Csn1s2b
Gm42209
2610028H24Rik
Ces5a
Hephl1
Foxl2os
Gm12199
Cd3e
Cntnap2
Pax3
Lrtm2
Cyp4f39
Tafa4
Caly
Gm16534
Gm12240
Gm48584
Mybphl
Gm28286
Sall1
Gm48199
Pvalb
Myh4
Gm48719
4933401D09Rik
Usp44
Calm4
C130021I20Rik
Epyc
Sorbs2os
Direction
Genotype
Sgcd-/-
Wild-type
MuscleType
EDL
Soleus
Z-score (VST)
1.8
0.9
0
-0.9
-1.8
Direction
Higher in EDL
Higher in Soleus
C
GO/KEGG enrichment — Soleus vs EDL in both genotypes
Muscle identity programs are compared within each genotype
Wild-type
Sgcd-/-
Higher in Soleus
Higher in EDL
ameboidal-type cell migration
Cytoskeleton in muscle cells - Mus musculus (house mouse)
lipid oxidation
fatty acid catabolic process
Citrate cycle (TCA cycle) - Mus musculus (house mouse)
epithelium migration
fatty acid oxidation
Non-alcoholic fatty liver disease - Mus musculus (house mouse)
fatty acid metabolic process
Regulation of actin cytoskeleton - Mus musculus (house mouse)
Diabetic cardiomyopathy - Mus musculus (house mouse)
epithelial cell migration
lipid modification
Chemical carcinogenesis - reactive oxygen species - Mus musculus (house mouse)
generation of precursor metabolites and energy
actin filament organization
regulation of actin filament-based process
energy derivation by oxidation of organic compounds
chemotaxis
Cytoskeleton in muscle cells - Mus musculus (house mouse)
Regulation of actin cytoskeleton - Mus musculus (house mouse)
Osteoclast differentiation - Mus musculus (house mouse)
Fc gamma R-mediated phagosome formation - Mus musculus (house mouse)
regulation of actin filament-based process
myeloid leukocyte migration
IgSF CAM signaling - Mus musculus (house mouse)
regulation of interleukin-6 production
leukocyte migration
wound healing
cell chemotaxis
myeloid leukocyte activation
actin filament organization
taxis
fatty acid metabolic process
leukocyte proliferation
muscle organ development
skeletal muscle organ development
GnRH signaling pathway - Mus musculus (house mouse)
multicellular organismal movement
Insulin signaling pathway - Mus musculus (house mouse)
Glioma - Mus musculus (house mouse)
musculoskeletal movement
macroautophagy
muscle cell differentiation
Oxytocin signaling pathway - Mus musculus (house mouse)
Autophagy - animal - Mus musculus (house mouse)
muscle cell development
striated muscle contraction
skeletal muscle tissue development
muscle system process
striated muscle cell differentiation
muscle contraction
Cytoskeleton in muscle cells - Mus musculus (house mouse)
striated muscle cell differentiation
striated muscle contraction
FoxO signaling pathway - Mus musculus (house mouse)
skeletal muscle contraction
Adrenergic signaling in cardiomyocytes - Mus musculus (house mouse)
glycogen metabolic process
GnRH signaling pathway - Mus musculus (house mouse)
muscle cell differentiation
Cytoskeleton in muscle cells - Mus musculus (house mouse)
Oxytocin signaling pathway - Mus musculus (house mouse)
Insulin signaling pathway - Mus musculus (house mouse)
monosaccharide metabolic process
glucose metabolic process
carbohydrate catabolic process
muscle system process
muscle contraction
muscle organ development
muscle cell development
− log10(padj)
30
20
10
D
Muscle fiber identity markers — Soleus vs EDL in both genotypes
VST-normalized expression (n = 3 per group); bars: mean +/- SD
Wild-type EDL
Wild-type Soleus
Sgcd-/- EDL
Sgcd-/- Soleus
VST normalized counts
Myh1
Myh2
Myh4
Myh7
Myl2
Myl3
Tnni1
Tnni2
Tnnc1
Tnnc2
Actn2
Actn3
