# Supplementary material for: Interferon-Associated Inflammatory Remodeling and Oxidative Metabolic Repression Define the Skeletal Muscle Transcriptomic Landscape of δ-Sarcoglycan Deficiency
Source: Biomolecules. 2026 Oct 17;16(10):1511. doi: 10.3390/biom16101511 (PMC13702233; doi:10.3390/biom16101511)
Supplement: Supplementary file 1 [file biomolecules-16-01511-s001.zip › Supplementary figures_/Supplementary figure S2.pdf]

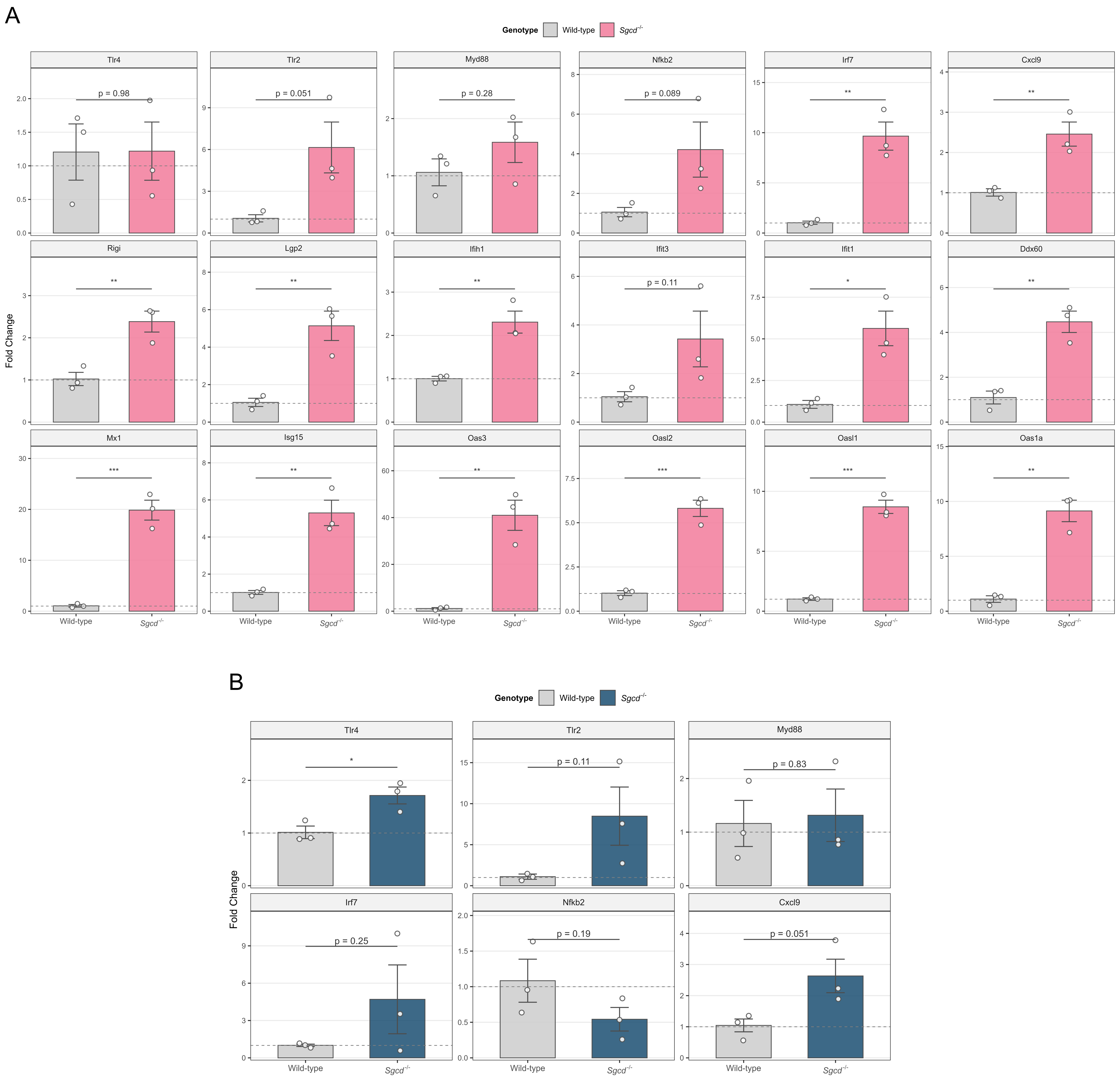
A
Genotype
Wild-type
Sgcd-/-
Tlr4
p = 0.98
Tlr2
p = 0.051
Myd88
p = 0.28
Nfkb2
p = 0.089
Irf7
**
Cxcl9
**
Rigi
**
Lgp2
**
Ifih1
**
Ifit3
p = 0.11
Ifit1
*
Ddx60
**
Mx1
***
Isg15
**
Oas3
**
Oasl2
***
Oasl1
***
Oas1a
**
Fold Change
Wild-type
Sgcd-/-
B
Genotype
Wild-type
Sgcd-/-
Tlr4
*
Tlr2
p = 0.11
Myd88
p = 0.83
Irf7
p = 0.25
Nfkb2
p = 0.19
Cxcl9
p = 0.051
Fold Change
Wild-type
Sgcd-/-
