# Supplementary material for: Interferon-Associated Inflammatory Remodeling and Oxidative Metabolic Repression Define the Skeletal Muscle Transcriptomic Landscape of δ-Sarcoglycan Deficiency
Source: Biomolecules. 2026 Oct 17;16(10):1511. doi: 10.3390/biom16101511 (PMC13702233; doi:10.3390/biom16101511)
Supplement: Supplementary file 1 [file biomolecules-16-01511-s001.zip › Supplementary figures_/Supplementary figure S3.pdf]

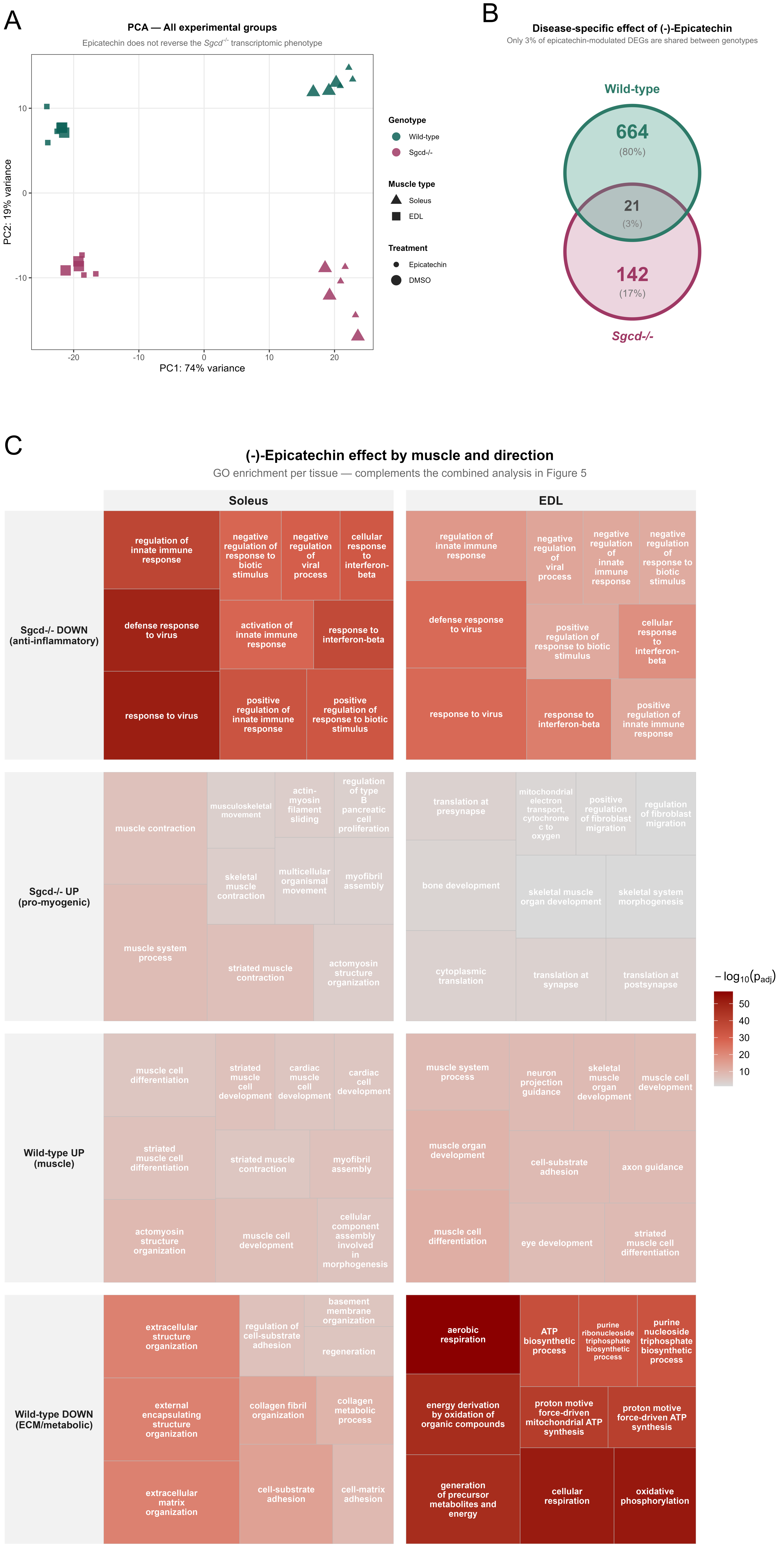

A
PCA — All experimental groups
Epicatechin does not reverse the Sgcd-/- transcriptomic phenotype
PC2: 19% variance
PC1: 74% variance
10
0
-10
-20
-10
0
10
20
Genotype
Wild-type
Sgcd-/-
Muscle type
Soleus
EDL
Treatment
Epicatechin
DMSO
B
Disease-specific effect of (-)-Epicatechin
Only 3% of epicatechin-modulated DEGs are shared between genotypes
Wild-type
664
(80%)
21
(3%)
142
(17%)
Sgcd-/-
C
(-)-Epicatechin effect by muscle and direction
GO enrichment per tissue — complements the combined analysis in Figure 5
Soleus
EDL
Sgcd-/- DOWN (anti-inflammatory)
regulation of innate immune response
negative regulation of response to biotic stimulus
negative regulation of viral process
cellular response to interferon-beta
defense response to virus
activation of innate immune response
response to interferon-beta
response to virus
positive regulation of innate immune response
positive regulation of response to biotic stimulus
regulation of innate immune response
negative regulation of viral process
negative regulation of innate immune response
negative regulation of response to biotic stimulus
defense response to virus
positive regulation of response to biotic stimulus
cellular response to interferon-beta
response to virus
response to interferon-beta
positive regulation of innate immune response
Sgcd-/- UP (pro-myogenic)
muscle contraction
musculoskeletal movement
actin-myosin filament sliding
regulation of type B pancreatic cell proliferation
skeletal muscle contraction
multicellular organismal movement
myofibril assembly
muscle system process
striated muscle contraction
actomyosin structure organization
translation at presynapse
mitochondrial electron transport, cytochrome c to oxygen
positive regulation of fibroblast migration
regulation of fibroblast migration
bone development
skeletal muscle organ development
skeletal system morphogenesis
cytoplasmic translation
translation at synapse
translation at postsynapse
Wild-type UP (muscle)
muscle cell differentiation
striated muscle cell development
cardiac muscle cell development
cardiac cell development
striated muscle cell differentiation
striated muscle contraction
myofibril assembly
actomyosin structure organization
muscle cell development
cellular component assembly involved in morphogenesis
muscle system process
neuron projection guidance
skeletal muscle organ development
muscle cell development
muscle organ development
cell-substrate adhesion
axon guidance
muscle cell differentiation
eye development
striated muscle cell differentiation
Wild-type DOWN (ECM/metabolic)
extracellular structure organization
regulation of cell-substrate adhesion
basement membrane organization
regeneration
external encapsulating structure organization
collagen fibril organization
collagen metabolic process
extracellular matrix organization
cell-substrate adhesion
cell-matrix adhesion
aerobic respiration
ATP biosynthetic process
purine ribonucleoside triphosphate biosynthetic process
purine nucleoside triphosphate biosynthetic process
energy derivation by oxidation of organic compounds
proton motive force-driven mitochondrial ATP synthesis
proton motive force-driven ATP synthesis
generation of precursor metabolites and energy
cellular respiration
oxidative phosphorylation
$-\log_{10}(p_{adj})$
50
40
30
20
10
